# Association between Suicide Rate and Human Development Index, Income, and the Political System in 46 Muslim-Majority Countries: An Ecological Study

**DOI:** 10.3390/ejihpe12070055

**Published:** 2022-07-09

**Authors:** S. M. Yasir Arafat, Marthoenis Marthoenis, Murad M. Khan, Mohsen Rezaeian

**Affiliations:** 1 Department of Psychiatry, Enam Medical College and Hospital, Dhaka 1340, Bangladesh; 2 Department of Psychiatry and Mental Health Nursing, Universitas Syiah Kuala, Banda Aceh 23111, Indonesia; marthoenis@unsyiah.ac.id; 3 Brain and Mind Institute, Aga Khan University, Karachi 74000, Pakistan; muradmk@gmail.com; 4 Epidemiology and Biostatistics Department, Occupational Environment Research Center, Rafsanjan University of Medical Sciences, Rafsanjan 7718175911, Iran; moeygmr2@yahoo.co

**Keywords:** Muslim country, suicide, human development index, income, political system

## Abstract

Very little has been researched assessing the relationship between the suicide rate and the ecological perspectives of the country, especially in the Muslim majority countries. We aimed to determine the association between suicide rate and the ecological parameters of 46 Muslim majority countries. We extracted the Muslim majority countries and their suicide rate, income distribution, distribution of the WHO region and continents, and Human Development Index (HDI). We assessed the correlation of the proportion of Muslim populations, the total population of the countries, number of suicides, continent, income group, political system, and HDI score with the suicide rate. The median suicide rate was 5.45 (IQR = 4.8); 2.9 (IQR = 4) in females and 7.45 (IQR = 8.2) in males per 100,000 population. The males had a significantly higher rate and the highest suicide rate was found in Africa. There are inverse associations between the total suicide rate, the rate in males, and females with HDI, and the income of the country. Furthermore, the suicide rate was significantly higher in countries with democratic systems compared to non-democratic countries. The findings suggest that ecological parameters may have an etiological role on suicides in Muslim countries where HDI and income are inversely associated with suicide rates.

## 1. Introduction

Suicide is a global public health issue. Suicide prevention is particularly challenging due to variable estimates, lack of research in many countries, and national priorities; this is further complicated by factors such as geographical location, income, the various statuses of several health indexes, and political systems that operate in a particular country [1,2]. Globally, about 703,000 people died by suicide in 2019, with an age-standardized rate of 9/100,000 for both sexes (12.6 for males, 5.4 for females per 100,000 population) comprising 1.3% of total deaths worldwide [1]. More than two-thirds of total suicides (77%) happened in low and middle-income countries (LMICs) where the prevention of suicide has been largely ignored [1].

Suicide is a highly complex phenomenon resulting from the multifactorial interaction of closely inter-linked factors such as gene and environment, stress-diathesis, distal and proximal factors, biopsychosocial, socio-cultural and religious factors [2,3]. As a result, any risk factor with a specific cause–effect association with suicide is difficult to identify [3]. Although psychiatric disorders have been identified as important risk factors for suicide consistently, the rate varies between Western countries and LMICs as studies from the former report rates around 90% whilst the LMICs show a much lower rate of mental disorders among suicides [3,4,5,6,7,8,9].

Islam is the second-largest religion in the world and there are approximately 50 countries and territories with Muslim majority population with about 1.9 billion adherents globally [10]. The majority of Muslim countries are situated in Asia and Africa with LMICs background. Suicide is prohibited in Islam, and in many Islamic countries it is still considered a criminal offense [11,12]. When compared to the global estimates, Muslim majority countries have a lower rate than others, indicating religious faith and practice as a protective factor for suicide [1,13,14,15]. However, several intricacies such as criminal status, stigma towards suicide, extreme dearth of research, poor political will (evidenced by low prevention activities), low-quality data, possible under-reporting and LMIC background have made the situation cloudy. One recent review identified that psychological autopsy studies have been conducted only in five Muslim countries (Bangladesh, Indonesia, Iran, Pakistan, and Turkey) of the world [9]. Studies have identified the association of lower suicide rates in countries with a higher number of Muslim populations [13,14]; however, the role of wider social determinants such as human development index (HDI), income category of the country, political system, and geographical position, have not been studied in the Muslim majority countries. In healthcare research, ecological perspective emphasizes both individual and contextual systems, and their interdependent relationship [16]. Additionally, due to the complex etiological association of suicide, ecological aspects could be an important area for suicide prevention. Without identifying the risk and protective factors, prevention of suicide would not be possible. Therefore, formulation and implementation of national suicide prevention strategies would not be effective unless risk factors are identified adequately. To fill the gap, we aimed to assess the association (if any) between suicide rate and ecological variables (population, HDI, income category, and political system) in 46 Muslim majority countries. The study would serve as a baseline exploration of the Muslim countries and hopefully lead to further studies in this area to inform suicide prevention strategies in Muslim majority countries. We considered the below-mentioned hypotheses.

**Hypothesis** **1** **(H1).** *The number of the Muslim population of a country is correlated with suicide rate.*

**Hypothesis** **2** **(H2).** *The total population of a country is correlated with suicide rate.*

**Hypothesis** **3** **(H3).** *The Muslim percentage of a country is correlated with suicide rate.*

**Hypothesis** **4** **(H4).** *The continent where a country situated is associated with suicide rate.*

**Hypothesis** **5** **(H5).** *The income group of a country is associated with suicide rate.*

**Hypothesis** **6** **(H6).** *The HDI score of a country is associated with suicide rate.*

**Hypothesis** **7** **(H7).** *The political system is associated with suicide rate.*

## 2. Materials and Methods

### 2.1. List of the Muslim Majority Countries

This was an ecological study where we collected secondary data from different openly available internationally recognized sources. We identified the Muslim majority countries from the *World Population Review* which reveals a list of 49 Muslim countries with a total number of country population, total number of Muslims, and proportion of Muslim population (Supplementary file S1) [10]. From the identified 49 countries, we excluded Western Sahara, Mayotte, and Palestine due to the unavailability of official suicide rates [1]; hence, our final analysis includes estimates of 46 Muslim majority countries (Table 1).

### 2.2. Suicide Rate, WHO Region, Continent, and Income

We extracted suicide rates (in total population, males, females), income distribution of the country, and distribution of the WHO region from the latest WHO report that includes the suicide estimate for 2019 [1]. We considered the age-standardized suicide rates, for all ages (per 100,000) in 2019 from the WHO report published in 2021. We identified Muslim countries from five WHO regions, namely, Eastern Mediterranean (EMR), Europe (EUR), Africa (AFR), Western Pacific Region (WPR), and South-East Asia Region (SEAR) (Table 1) [1]. No Muslim country was identified from the region of the Americas (AMR) among the selected 46 countries. We selected the continent of the countries based on the available list from the *World Population Review* [17]. Among the 46 countries, 18 were identified from Africa, 26 from Asia, and the other 2 were from Europe while considering the continent-wise distribution of the countries. We took the recent classification provided by the *World Bank* (WB) while classifying the countries based on income [18]. As per the WB category, we divided the countries into high-income (HI), low-income (LI), lower-middle-income (LMI), and upper-middle-income (UMI) (Table 1) [18].

### 2.3. Human Development Index (HDI) 

We extracted the HDI from the Global Human Development Indicators of 2020 [19]. We extracted the index value against each country and determined the association with suicide rate; it ranges from 0.39 for Niger to 0.89 for United Arab Emirates (Table 1). We considered the *United Nation Development Program (UNDP)* provided the definition of HDI mentioning HDI as the “summary measure of average achievement in key dimensions of human development: a long and healthy life, being knowledgeable and have a decent standard of living” [20].

### 2.4. Procedure 

To test the hypotheses, we extracted the study (mentioned earlier) variables from readily available secondary sources. We extracted the data into *Microsoft Excel* software, version 2010. Two authors individually extracted the data which were cross-checked by a third individual. The variables were so well-defined that there were no points of ambiguity except the classification of countries based on political ideology. In case of any ambiguity, the whole team was involved to make the decision. 

### 2.5. Statistical Analysis

Statistical analysis was done in Stata version 13. Before performing the statistical analysis, we checked the assumptions for a parametric test. The majority of the test variables were not normally distributed; therefore, non-parametric tests were used. The correlation between the number of Muslim populations, total population of the countries, Muslim percentages, number of suicides, and HDI score and suicide rates was tested using Spearman’s correlation. The association between the continent and income group with suicide rate was tested using the Kruskal Wallis test. The association between the political system and the suicide rate was tested using the Mann–Whitney U test. The difference in median suicide rate between males and females was observed using the Wilcoxon rank-sum test. As the countries pertain to the three continents (Africa, Asia, and Europe), the rest of the continents were excluded from the analysis. 

## 3. Results

### 3.1. Suicide Rate in Muslim Countries 

The list of the 46 Muslim countries, the total population, the total number of Muslim populations, the proportion of Muslim populations, the total number of suicides, rate of the total, male, and female suicide, income group, HDI score, geographical location of the countries (continent & WHO region), and political systems were extracted from openly available recognized sources (Table 1). The overall median suicide rate was 5.45 (IQR = 4.8) for the total population, 2.9 (IQR = 4) for females, and 7.45 (IQR = 8.2) for males per 100,000 population in the Muslim countries in 2019. The suicide rate was therefore significantly higher in males than females (Wilcoxon Rank Sum Test; *p* < 0.0001). 

### 3.2. Association between Suicide Rate and Ecological Variables 

Four ecological variables i.e., the residing continent of the country, income group, HDI score, and political system were associated with total suicide rate, the suicide rates in males, and females (Table 2). A higher suicide rate (total, male, and female populations) was noted in African Muslim countries, followed by Asian countries while the two European Muslim countries revealed the lowest suicide rate (Table 2). These differences were statistically significant (Table 2). 

**Figure 1:**
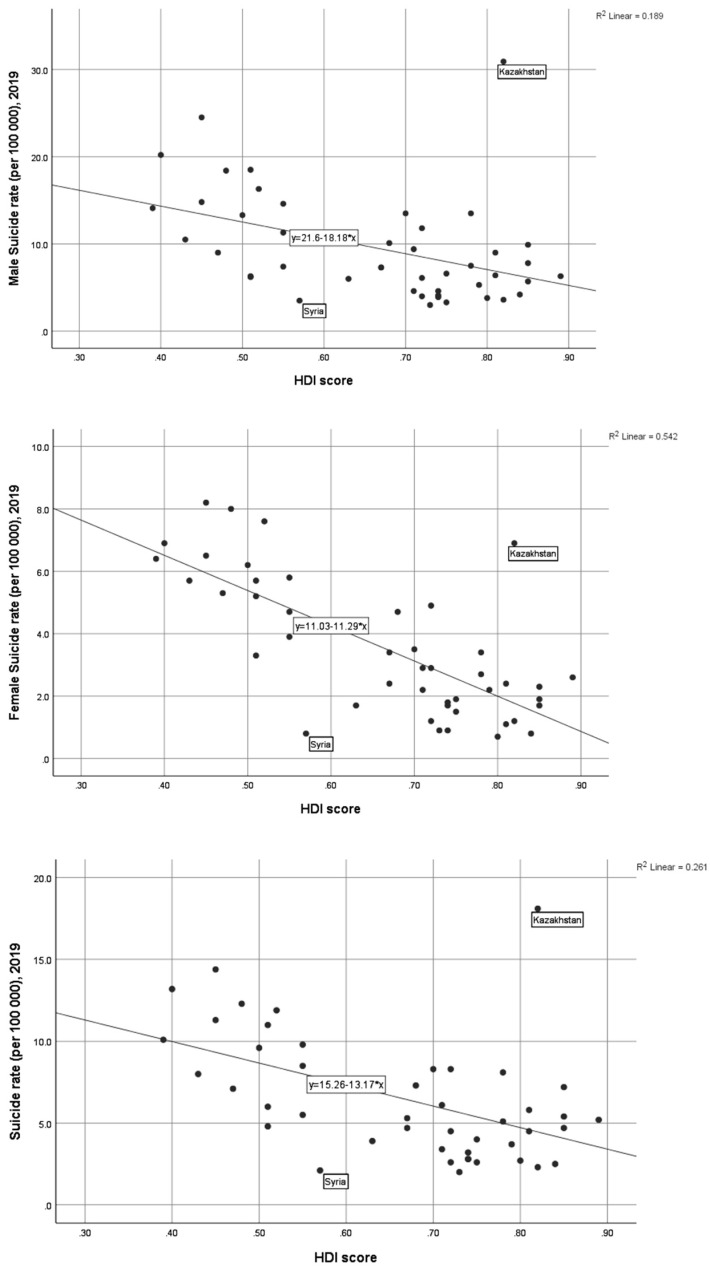
Scatter diagram showing the association between suicide rate (total, male, and female) and HDI.

There are inverse associations between suicide rates and HDI score, whilst female suicide rate seems to be more inversely associated with HDI compared to male suicide rate where Syria and Kazakhstan were found as two outliers (Figure 1; Table 2). The finding of the inverse relationship between HDI score and suicide rate suggests that the Muslim countries with a higher HDI score revealed a lower suicide rate (total, male, and female) except Syria and Kazakhstan (Figure 1; Table 2). The inverse relationships between suicide rates and HDI scores were statistically significant (Table 2). 

Likewise, the HDI score, significantly higher suicide rates (total, male, and female) were found in LI countries, followed by LMI, UMI, and HI countries. The findings suggest that countries with poor socioeconomic backgrounds revealed a higher suicide rate among the 46 Muslim countries (Table 1 and Table 2). 

Furthermore, significantly higher suicide rates (total, male, and female) were found in countries with a democratic political system compared to non-democratic countries. The non-democratic countries included monarchy, military, and sharia-based countries. The findings suggest the association of the political system with suicide as a wider ecological parameter in Muslim countries (Table 2). 

## 4. Discussion

### 4.1. Major Findings 

This study aimed to determine associations between suicide rate and ecological variables (total population, proportion of Muslim population, HDI, income category, residing continent of the Muslim country, and political system) in 46 Muslim majority countries. We identified Muslim majority countries and their suicide rate, income distribution of the country, distribution of the WHO region and continents, and HDI and tested the ecological relationship. The analysis found the suicide rate in the Muslim majority countries was lower than the global average in 2019, significantly higher among males, and in the African Muslim countries (Table 1 and Table 2). The study also revealed that there are inverse associations between the total rate, the rate in males, and the rate in females with HDI, and the income of the country.

The rate of suicide in the Muslim majority countries has been found lower when compared to the global rates with few exceptions such as some African Muslim countries Burkina Faso, Chad, Guinea, and Somalia (Table 1) [1]. The only Asian Muslim country, Kazakhstan, had a higher rate. Whether this can be attributed to the socialist political system during the association with Union of Socialist Soviet Republics needs further exploration [18]. The finding of gender variation of suicide rate has no global exception where males die by suicide more than females; however, available evidence suggests that the females have a comparatively higher proportion in LMICs [1] indicating females may be more vulnerable in the LMICs than in the HI countries. Although the current study does not reveal a lower male to female ratio it reveals a steeper association between female suicide rate and HDI score when compared to the total suicide and the male suicide rate (Figure 1); however, several confounders such as under-reporting and misclassification of suicides, data quality, and lack of suicide research in Muslim countries should be considered while generalizing the findings [21]. There are clear variations in the suicide rate among the countries of the Middle-East and Africa. Additionally, the lifestyles of Muslims in Africa vary when compared to the Middle-East countries. Further studies are warranted to identify the basis of the associations; whether they are due to cultural factors specifically, due to economic factors, or interaction between the factors needs further exploration. 

Our findings show inverse associations between HDI, income, and suicide rates that explain the role of socio-economic position and suicide. A similar association was noted in previous studies in the United States, Japan, Australia, and England while a positive correlation was found in Italy [22,23,24,25,26]. The positive correlation is also reported in a global ecological study assessing data of 91 countries [27]; however, one systematic review of South Asian LMICs identified that lower socio-economic status is likely to increase suicidal behavior [28]. Another recent study covering 46 Muslim countries identified the association between HDI and age-standardized suicide rates of suicide [13]. 

The inverse association between suicide rate and HDI could also be investigated in each component of HDI; life expectancy, access to education and standard of living. Suicide rate is significantly responsible for a country’s life expectancy. Increased suicide rate reduces the country’s life expectancy, while eliminating suicide as the cause of death increase life expectancy at birth [29,30]; likewise, another study identified that the suicide rate tends to be higher in lower-income groups [31]. On the other hand, suicide tends to be higher among those with a higher level of education revealed by a study assessing the level of education among suicide deaths, yet, the finding that low educational attainment inflates the risk of suicide has also been shown in other studies [32,33]. The inconsistency between suicide rates and the HDI components in the studies calls for further investigations, considering each HDI component specifically over the years.

### 4.2. Implications

The findings have several implications. The possible protective factors that could explain the lower rate of suicide in the Muslim majority countries should be identified and strengthened while formulating suicide prevention strategies. On the other hand, local, regional, and international agencies should take initiatives to improve the quality of data for suicide reporting, avoiding the under-reporting and misclassification of suicides, reducing the stigma, and decriminalization of suicide in the legal system [2,11,12,20]. Geographical variation of suicide i.e., higher rate in African Muslim countries than in Asian ones highlights the possible effect of socio-economic background and warrants further investigations. The findings clearly depict the role of HDI and income on suicides indicating the effect of the wider social and ecological variable on emotions, behavior and mental distress and help situate suicidal behavior within the wider environmental ecosystems (micro-, meso-, exo-, macro-, and chrono-system) [34]; moreover, it reinforces the complex interaction of risk factors for suicide. Prevention strategies should focus on improving the overall quality of life, income, and HDI. A stable political system based on democratic principles ensures the transparency and accountability that creates standard reporting of suicide data. 

### 4.3. What Is Already Known

The average rate of suicide is lower in the Muslim majority countries when compared to the global average. The Islamic religion has a strong influence on suicidal behaviors; however, risk factors for suicide and suicide prevention have been under-prioritized, especially in Muslim majority LMICs.

### 4.4. What This Study Adds

This study identified and determined the relationship between the wider socio-demographic variable with the suicide rates in the Muslim majority countries. African Muslim majority countries had a significantly higher suicide rate when compared to Asian and European Muslim countries. The suicide rate is inversely correlated with the HDI and income of the country in all Muslim countries with two exceptions (Syria and Kazakhstan). The suicide rate is higher in the Muslim majority countries with democratic political systems when compared to others.

### 4.5. Strength of the Study

This is one of the first studies determining the relationship between suicide rate and wider social determinants of health in Muslim majority countries.

### 4.6. Limitations of the Study

Several important limitations should be considered while implementing the study results. Firstly, we extracted data from available global estimates of 46 Muslim majority countries. Even though the well-designed definition facilitates comparison, individual country-wise interpretation is limited. Secondly, the quality of suicide data has been noticed to be different based on the HDI score and income of the country. Thirdly, the legal status of suicide has varied between countries, which could be a potential source of bias in suicide reporting. Fourthly, the quality of the suicide reporting system, under-reporting, and misclassification of suicide are potential variables to consider while generalizing the study results. Fifthly, there might have been recent changes in the political system of countries such as democratic government may have lost the characteristics of standard democracy, whilst autocratic countries may have shifted to democracy.

### 4.7. Future Lines of Research

The study identified some associations only without any exploration regarding the direction of the association. Further studies are warranted with the aim to assess the direction of the associations. Additionally, studies assessing the role of individual components of HDI could be tested. Special attention could be given to finding out the reasons for the high suicide rates in democratic Muslim countries. More importantly, religion-sensitive prevention strategies should be tested and identified for the Muslim majority countries. We recommend more attention to research on suicide and suicidal behavior in Muslim countries, country- and region-wise to overcome the shortage of evidence.

## 5. Conclusions

Our study is one of the first studies assessing the relationship between suicide rates and ecological parameters of the Muslim majority countries using available global estimates; it identified significant associations between suicide rate and geography i.e., continent, socioeconomic status i.e., income category, and HDI, and political system. The findings suggest that ecological parameters have an attributing role to the suicide rate of Muslim countries where HDI and income are inversely associated with suicide rate; however, because of multi-factorial causation and complexity of the phenomenon a cautious interpretation is needed regarding the causal role of the ecological parameters and further longitudinal studies are warranted to reveal the association more precisely.

## Figures and Tables

**Table 1:** Extracted country-wise rates.

SN	Country	Muslim Population (2021) (Million)	Total Population (2021) (Million)	Muslim %	Suicide Rate (per 100,000), 2019	Male Suicide Rate (per 100,000), 2019	Female Suicide Rate (per 100,000), 2019	Number of Suicides	Continent	WHO Region	Income Group	HDI Score	Political System
1	Afghanistan	34.84	39.84	99.6	6	6.2	5.7	1573	Asia	EMR	LI	0.51	Sharia
2	Albania	1.8	2.87	58.8	3.7	5.3	2.2	125	Europe	EUR	UMI	0.79	Democracy
3	Algeria	41.24	44.62	99	2.6	3.3	1.9	1072	Africa	AFR	LMI	0.75	Democracy
4	Azerbaijan	9.74	10.22	96.9	4	6.6	1.5	411	Asia	EUR	UMI	0.75	Democracy
5	Bahrain	1.06	1.75	73.7	7.2	9.9	2.3	145	Asia	EMR	HI	0.85	Monarchy
6	Bangladesh	153.7	166.3	90.4	3.9	6	1.7	5998	Asia	SEAR	LMI	0.63	Democracy
7	Bosnia and Herzegovina	1.96	3.26	50.7	8.2	13.5	3.4	361	Europe	EUR	UMI	0.78	Democracy
8	Brunei	0.36	0.44	78.8	2.5	4.2	0.8	12	Asia	WPR	HI	0.84	Monarchy
9	Burkina Faso	12.14	21.5	61.5	14.4	24.5	6.5	1521	Africa	AFR	LI	0.45	Democracy
10	Chad	9.18	16.91	58	13.2	20.2	6.9	1027	Africa	AFR	LI	0.4	Democracy
11	Comoros	0.81	0.89	98.3	8.5	11.3	5.8	46	Africa	AFR	LMI	0.55	Democracy
12	Djibouti	0.86	1	97	11.9	16.3	7.6	94	Africa	EMR	LMI	0.52	Democracy
13	Egypt	87.50	104.26	92.35	3.4	4.6	2.2	3022	Africa	EMR	LMI	0.71	Military
14	Gambia	2	2.49	95.7	9.6	13.3	6.2	113	Africa	AFR	LI	0.5	Democracy
15	Guinea	10.56	13.5	89.1	12.3	18.4	8	892	Africa	AFR	LI	0.48	Democracy
16	Indonesia	229	276.36	87.2	2.6	4	1.2	6544	Asia	SEAR	UMI	0.72	Democracy
17	Iran	82.5	85.03	99.4	5.1	7.5	2.7	4334	Asia	EMR	UMI	0.78	Democracy
18	Iraq	38.47	4.12	95.7	4.7	7.3	2.4	1418	Asia	EMR	UMI	0.67	Democracy
19	Jordan	10.17	10.27	97.2	2	3	0.9	165	Asia	EMR	UMI	0.73	Monarchy
20	Kazakhstan	13.16	18.99	70.2	18.1	30.9	6.9	3256	Asia	EUR	UMI	0.82	Democracy
21	Kuwait	2.18	0.43	74.6	2.7	3.8	0.7	122	Asia	EMR	HI	0.8	Monarchy
22	Kyrgyzstan	4.68	6.63	80	8.3	13.5	3.5	474	Asia	EUR	LMI	0.7	Democracy
23	Lebanon	3.52	6.77	57.7	2.8	3.9	1.7	190	Asia	EMR	UMI	0.74	Democracy
24	Libya	6.55	6.96	97	4.5	6.1	2.9	304	Africa	EMR	UMI	0.72	Democracy
25	Malaysia	16.32	32.78	61.3	5.8	9	2.4	1823	Asia	WPR	UMI	0.81	Democracy
26	Maldives	0.39	0.54	98.4	2.8	4.1	0.9	15	Asia	SEAR	UMI	0.74	Democracy
27	Mali	17.51	20.86	95	8	10.5	5.7	806	Africa	AFR	LI	0.43	Democracy
28	Mauritania	3.84	4.78	100	5.5	7.4	3.9	141	Africa	AFR	LMI	0.55	Democracy
29	Morocco	37.93	37.34	99	7.3	10.1	4.7	2617	Africa	EMR	LMI	0.68	Monarchy
30	Niger	21.1	25.13	98.3	10.1	14.1	6.4	1227	Africa	AFR	LI	0.39	Democracy
31	Oman	2.43	5.22	85.9	4.5	6.4	1.1	241	Asia	EMR	HI	0.81	Monarchy
32	Pakistan	200.4	225.2	96.5	9.8	14.6	4.7	19,331	Asia	EMR	LMI	0.55	Democracy
33	Qatar	1.57	2.93	77.5	4.7	5.7	1.7	165	Asia	EMR	HI	0.85	Monarchy
34	Saudi Arabia	31.88	35.34	97.1	5.4	7.8	1.9	2046	Asia	EMR	HI	0.85	Monarchy
35	Senegal	15.11	17.2	96.1	11	18.5	5.2	978	Africa	AFR	LMI	0.51	Democracy
36	Sierra Leone	6.07	8.14	78.6	11.3	14.8	8.2	521	Africa	AFR	LI	0.45	Democracy
37	Somalia	10.98	16.36	99.8	14.7	22.8	7.1	1219	Africa	EMR	LI		Democracy
38	Sudan	39.59	44.91	97	4.8	6.3	3.3	1644	Africa	EMR	LI	0.51	Authoritarian
39	Syria	16.7	18.28	93	2.1	3.5	0.8	333	Asia	EMR	LI	0.57	Authoritarian
40	Tajikistan	7.62	9.75	96.7	5.3	7.3	3.4	399	Asia	EUR	LI	0.67	Democracy
41	Tunisia	11.19	11.94	99.8	3.2	4.6	1.8	383	Africa	EMR	LMI	0.74	Democracy
42	Turkey	79.85	85.04	99.2	2.3	3.6	1.2	2003	Asia	EUR	UMI	0.82	Democracy
43	Turkmenistan	4.83	6.12	93.3	6.1	9.4	2.9	337	Asia	EUR	UMI	0.71	Democracy
44	United Arab Emirates	4.62	9.99	76	5.2	6.3	2.6	628	Asia	EMR	HI	0.89	Monarchy
45	Uzbekistan	26.55	33.94	96.5	8.3	11.8	4.9	2653	Asia	EUR	LMI	0.72	Democracy
46	Yemen	27.78	30.49	99.1	7.1	9	5.3	1699	Asia	EMR	LI	0.47	Democracy

HI = high-income, LI = low-income, LMI = lower-middle-income, UMI = upper-middle-income, EMR = Eastern Mediterranean, EUR = Europe, AFR = Africa, WPR = Western Pacific Region, SEAR = South-East Asia Region.

**Table 2:** Association between suicide rates with populations, continent, income of the country, HDI score, and political system.

	Total Suicide Rate		Suicide Rate in Male		Suicide Rate in Female	
	Statistics	*p*-Value	Statistics	*p*-Value	Statistics	*p*-Value
Muslim population	rs = −0.06	0.64	rs= −0.05	0.7	rs = 0.03	0.83
Total population	rs = 0.009	**0.001**	rs = −0.003	0.98	rs = 0.08	0.56
Muslim percentage	rs = −0.08	0.55	rs = −0.12	0.41	rs = 0.07	0.61
Number of suicide	rs = 0.18	0.22	rs = 0.19	0.18	rs = 0.20	0.17
Continent	X^2^(2) = 8.1	**0.01**	X^2^ (2) = 6.74	**0.03**	X^2^ (2) = 15.01	**0.001**
Income group	X^2^(2) = 10.89	**0.01**	X^2^(2) = 8.02	**0.04**	X^2^(2) = 19.6	**0.001**
HDI Score	rs = −0.52	**0.001**	rs= −0.47	**0.001**	rs = −0.67	**0.001**
Political system *	U = 1679	**0.015**	U = 1679	**0.001**	U = 1678	**0.001**

* considered democratic and others, Bold indicates *p* < 0.05.

## Data Availability

The data that support the findings of this study are mentioned in the manuscript.

## Supplementary Materials

The following supporting information can be downloaded at: https://www.mdpi.com/article/10.3390/ejihpe12070055/s1.
