# Supplementary material for: Association between Suicide Rate and Human Development Index, Income, and the Political System in 46 Muslim-Majority Countries: An Ecological Study
Source: Eur J Investig Health Psychol Educ. 2022 Jul 9;12(7):754–64. doi: 10.3390/ejihpe12070055 (PMC9318836; doi:10.3390/ejihpe12070055)
Supplement: Supplementary file 1 [file ejihpe-12-00055-s001.zip › ejihpe-1765828-supplementary.pdf]

**Supplementary 1: List of the Muslim countries**

| country | Muslim Population | pop2021 | Muslim % |
|---|---|---|---|
| Afghanistan | 34836014 | 39835.428 | 99.6 |
| Albania | 1797645 | 2872.933 | 58.8 |
| Algeria | 41240913 | 44616.624 | 99 |
| Azerbaijan | 9735074 | 10223.342 | 96.9 |
| Bahrain | 1063239 | 1748.296 | 73.7 |
| Bangladesh | 153700000 | 166303.498 | 90.4 |
| Bosnia and Herzegovina | 1955084 | 3263.466 | 50.7 |
| Brunei | 355045 | 441.532 | 78.8 |
| Burkina Faso | 12141769 | 21497.096 | 61.5 |
| Chad | 9183207 | 16914.985 | 58 |
| Comoros | 807204 | 888.451 | 98.3 |
| Djibouti | 857496 | 1002.187 | 97 |
| Egypt | 87500000 | 104258.327 | 92.35 |
| Gambia | 2002743 | 2486.945 | 95.7 |
| Guinea | 10563171 | 13497.244 | 89.1 |
| Indonesia | 229000000 | 276361.783 | 87.2 |
| Iran | 82500000 | 85028.759 | 99.4 |
| Iraq | 38465864 | 41179.35 | 95.7 |
| Jordan | 10165577 | 10269.021 | 97.2 |
| Kazakhstan | 13158672 | 18994.962 | 70.2 |
| Kuwait | 2175684 | 4328.55 | 74.6 |
| Kyrgyzstan | 4679436 | 6628.356 | 80 |
| Lebanon | 3519743 | 6769.146 | 57.7 |
| Libya | 6551871 | 6958.532 | 97 |
| Malaysia | 16318355 | 32776.194 | 61.3 |
| Maldives | 386193 | 543.617 | 98.4 |
| Mali | 17508398 | 20855.735 | 95 |
| Mauritania | 3840429 | 4775.119 | 100 |
| Mayotte | 253439 | 279.515 | 97 |
| Morocco | 37930989 | 37344.795 | 99 |
| Niger | 21101926 | 25130.817 | 98.3 |
| Oman | 2427000 | 5223.375 | 85.9 |
| Pakistan | 200400000 | 225199.937 | 96.5 |
| Palestine | 4298000 | 5222.748 | 97.5 |
| Qatar | 1566786 | 2930.528 | 77.5 |
| Saudi Arabia | 31878000 | 35340.683 | 97.1 |
| Senegal | 15112721 | 17196.301 | 96.1 |
| Sierra Leone | 6067706 | 8141.343 | 78.6 |

| | | | |
|---|---|---|---|
| Somalia | 10978000 | 16359.504 | 99.8 |
| Sudan | 39585777 | 44909.353 | 97 |
| Syria | 16700000 | 18275.702 | 93 |
| Tajikistan | 7621700 | 9749.627 | 96.7 |
| Tunisia | 11190000 | 11935.766 | 99.8 |
| Turkey | 79850000 | 85042.738 | 99.2 |
| Turkmenistan | 4830000 | 6117.924 | 93.3 |
| United Arab Emirates | 4615081 | 9991.089 | 76 |
| Uzbekistan | 26550000 | 33935.763 | 96.5 |
| Western Sahara | 599633 | 611.875 | 99.4 |
| Yemen | 27784498 | 30490.64 | 99.1 |

Source: World Population Review, nd. Muslim Majority Countries 2021. (accessed on 15 June 2021). https://worldpopulationreview.com/country-rankings/muslim-majority-countries
